# The influence of self‐management, anxiety and depression on chronic eczema‐related quality of life

**DOI:** 10.1002/ski2.106

**Published:** 2022-03-07

**Authors:** Laura Standen, Gulcan Garip

**Affiliations:** ^1^ College of Health, Psychology & Social Care University of Derby Derby UK

## Abstract

Chronic eczema, a persistent inflammatory skin condition, affects 1 in 12 adults in the United Kingdom and negatively influences quality of life. Self‐management can potentially influence chronic conditions, such as eczema, reducing symptoms and positively influencing quality of life; however, there is a lack of public education for eczema. Anxiety and depression negatively influence quality of life, and frequently present alongside eczema. Psychological interventions for anxiety and depression have shown to be effective for eczema‐related quality of life. This study aimed at examining the relationship between self‐management, anxiety, and depression, on quality of life in individuals with chronic eczema. The main hypothesis proposed that anxiety and depression reduce the influence of eczema self‐management on quality of life, potentially as individuals might be less likely to support their eczema treatment when experiencing symptoms of anxiety and depression. A cross‐sectional design and opportunistic sampling were used to analyse the data from 77 participants who responded to four self‐report scales to assess self‐management understanding, anxiety, depression and quality of life in participants with chronic eczema. Data were analysed by a hierarchical multiple linear regression to assess the variance contributed by each variable added to the model. Results from this sample shows a trend whereby self‐management of eczema contributes less variance to quality of life alongside comorbid anxiety and depression; this suggests that self‐management has reduced influence on eczema‐related quality of life when anxiety and depression are present. Furthermore, low self‐management, high anxiety, and high depression significantly negatively influence quality of life. The three variables combined accounted for 41.1% of variance in quality of life scores, suggesting anxiety, depression and self‐management influence quality of life in the sample. Self‐management, anxiety, and depression significantly influence eczema‐related quality of life, and participants who reported comorbid anxiety and depression were more likely to report lower levels of self‐management in this sample.

1

1 **What is already known about this topic?**

- Eczema significantly negatively influences quality of life. Self‐management of chronic conditions positively influences quality of life; lack of self‐management education negatively influences eczema symptoms.
- Eczema can increase the likelihood of anxiety and depression, while anxiety is more frequently associated with eczema. Both anxiety and depression negatively influence eczema‐related quality of life.

2 **What does this study add?**

- This study looks at the combined influence of self‐management, anxiety and depression on eczema‐related quality of life. Furthermore, this study looks at the influence of anxiety and depression on the self‐management of eczema in relation to quality of life.
- It is important to consider for anxiety and depression alongside offering self‐management education for eczema patients in primary care settings.

## 2 INTRODUCTION

Chronic eczema affects 1 in 12 adults in the United Kingdom, sometimes lasting a lifetime, causing unbearable physical and psychological symptoms^1^ that negatively impact quality of life,^2^, ^3^, ^4^, ^5^ a vital part of holistic health care.^6^ Chronic eczema is a persistent inflammatory skin condition that can result in patches of dry, cracked, red and inflamed skin that can cause itch, pain and sleep disturbance,^7^ as well as stigma, discrimination and isolation.^8^ Chronic eczema includes different kinds of eczema including atopic, contact, neurodermatitis, dyshidrotic, nummular, seborrhoeic and stasis eczema. People with eczema report significantly lower levels of quality of life,^9^, ^10^ understood in this study as the individual's understanding of their cultural position, expected standards, personal aims and concerns.^11^

### 2.1 Self‐management

Self‐management of eczema is understood as knowledge of eczema and treatments, appointment attendance and shared decision‐making with healthcare professionals, symptom monitoring, and healthy lifestyle choices.^12^ Self‐management of chronic conditions, such as eczema, improves patient wellbeing and quality of life.^13^, ^14^, ^15^, ^16^ Effective self‐management positively influences quality of life for chronic conditions such as asthma and diabetes.^17^ Eczema requires high self‐management^18^; however, eczema research presents mixed findings.^19^, ^20^

### 2.2 Anxiety

Anxiety includes nervousness, fearfulness, and raised heart and breathing rates.^21^ Anxiety is more frequently associated with eczema than depression,^4^ and at significantly higher levels than in control populations.^2^, ^3^ Anxiety is strongly associated with poorer quality of life,^21^, ^22^ possibly due to anxiety resulting in a lack of treatment motivation.^2^, ^23^ Psychological interventions for anxiety are effective for eczema‐related quality of life.^8^

### 2.3 Depression

Depression includes fatigue, hopelessness, lack of motivation and loss of interest in previously joyful activities.^24^ Furthermore, depression can influence healthy lifestyle behaviours and medical treatment adherence.^25^ Depression negatively influences quality of life,^22^, ^26^ and physical eczema symptoms might increase the risk of depression, potentially decreasing quality of life.^27^ The association between depression and eczema^7^, ^27^ and the influence on quality of life^10^ have produced mixed findings. A meta‐analysis^27^ found a significant correlation between depression and eczema, suggesting an increased risk of depression.

## 3 AIMS AND OBJECTIVES

The aims of this study are to assess whether self‐management, anxiety, and depression influence eczema‐related quality of life. Furthermore, the analysis aims to establish whether anxiety and depression reduce the influence of self‐management on quality of life. It is anticipated that the presence of anxiety and depression potentially reduce self‐management variance and influence quality of life.

### 3.1 Hypotheses

1. High levels of self‐management, and low levels of anxiety and depression, together positively influence quality of life in individuals with chronic eczema.
2. Low levels of self‐management, and high levels of anxiety and depression, together negatively influence quality of life in individuals with chronic eczema.
3. High levels of anxiety and depression reduce the influence of self‐management on quality of life in individuals with chronic eczema.

## 4 MATERIALS AND METHODS

### 4.1 Design

The cross‐sectional design of this correlational study aimed to establish whether the variables of self‐management, anxiety and depression influence eczema‐related quality of life, using a hierarchical multiple linear regression across three models.

### 4.2 Recruitment and participants

A participant invitation was posted on social media (Facebook and Twitter), public survey‐sharing websites, and the University of Derby research sharing scheme, whereby members of the public who self‐declared a diagnosis of chronic eczema could voluntarily participate in the survey. A total of 77 participants were opportunistically recruited in this manner. Eligible participants were aged 18+ with a self‐reported current diagnosis of chronic eczema, not from a vulnerable population (e.g., mental disabilities), and not currently receiving treatment for anxiety or depression. These criteria were self‐declared by each participant as a prerequisite for continuing with the online survey, but no further evidence other than the self‐declared participant statement of a chronic eczema diagnosis was required. A total of 125 responses were collected; 27 partial responses were excluded and a further 21 responses failed to satisfy the attention check question. The final number of participants was 77. Of these participants, 69% identified as female (*n* = 53; male = 31%, *n* = 24) and 77% selected the 18–29 age bracket (*n* = 59). Participants identified 66% as White (*n* = 51), and 52% of participants had university education (*n* = 40). Current health status reported 61% ‘good’ (*n* = 47). Table 1 presents complete demographics.

**TABLE 1:** Demographics of participants

	Frequency	Percentage (%)
Sex		
Female	53	68.83
Male	24	31.17
Age		
18–29	59	76.62
30–39	13	16.88
40–49	3	3.90
50–59	2	2.60
Education		
Primary school	1	1.30
Secondary school	14	18.18
University	40	51.95
Postgraduate	21	27.27
Prefer not to say	1	1.30
Health		
Very good	12	15.58
Good	47	61.04
Neither poor nor good	11	14.29
Poor	7	9.09

### 4.3 Procedure

Participants completed the total 65 questions, with each of the four measures presented in randomized order to avoid order bias. The survey was created, and accessed and completed by participants in Qualtrics on a computer or mobile browser, and the survey lasted 7–25 min as recorded by qualtrics.

### 4.4 Measures

#### 4.4.1 Partners in Health (PIH) scale^12^

Assessment for self‐management of chronic conditions, such as eczema, comprising 11 questions on a Likert scale (0 (very good)–8 (very poor). The reliability of the scale is good (*α* = 0.88). Construct validity established three factors—core self‐management (variance = 46%); condition knowledge (variance = 13%); symptom monitoring, (variance = 9%). An example question is ‘My knowledge of my condition is…’.

#### 4.4.2 General Anxiety Disorder 7 (GAD7) scale^28^

Assessment for anxiety, comprising seven questions on a Likert scale (0 (not at all)–3 (nearly every day). Scale sensitivity is 89% and specificity is 82% for generalized anxiety disorder. An example question is ‘Over the last 2 weeks, how often have you been bothered by the following problems? … Trouble relaxing’.

#### 4.4.3 Centre for Epidemiologic Studies Depression (CES‐D) scale^29^

Assessment for symptoms of depression, comprising 20 questions presented on a Likert scale (0 (rarely or none of the time)–3 (all of the time). The scale has high internal consistency reliability (0.91). An example question is ‘During the past week… my sleep was restless’.

#### 4.4.4 World Health Organization Quality of Life Bref (WHOQOL‐BREF) scale^30^

Assessment for quality of life, considering four domains (physical health; psychological; social relationships and environment), and comprising 26 questions on a Likert scale (1 (very poor/not at all)–5 (very good/completely). The four domains have total possible scores of 35, 30, 15 and 40, respectively. Raw scores are transformed onto a 0–100 scale for direct comparison, and the mean score of the four domains used as a single final score for each participant. An example question is ‘How satisfied are you with yourself?’

### 4.5 Data analysis

The data were analysed using Excel 2016 and SPSS 26. Sample data were compared to population averages. Hierarchical multiple linear regression added each variable one by one across three models (Model 1: self‐management; Model 2: anxiety; Model 3: depression) to investigate variance contributed.^31^

### 4.6 Ethical considerations

Ethical approval was granted by the University of Derby Research Ethics Committee. All ethical considerations were taken into account with regards the British Psychological Society (BPS) Standards and Requirements,^32^ the BPS Ethics Guidelines for Internet‐Mediated Research,^33^ the University of Derby Code of Ethics,^34^ and General Data Protection Regulation.^35^

## 5 RESULTS

The aims of this study were to assess whether self‐management, anxiety and depression influence quality of life alongside chronic eczema, and whether anxiety and depression reduce the influence of self‐management on quality of life. Initially, means and standard deviations (see Table 2) were analysed. Assumptions of normality were met and data were accepted as parametric.

**TABLE 2:** Means (*M*), standard deviations (*SD*), 95% confidence intervals (CI), skewness and kurtosis with standard errors (SE) for CES‐D, GAD7, PIH and WHOQOL‐BREF scores

Variable	*M* (*SD*)	95% CI lower	95% CI upper	Skewness (SE)	Kurtosis (SE)	K–S test (sig.)	S–W test (sig.)	*N*
CES‐D	22.49 (11.27)	19.94	25.05	0.41 (0.27)	−0.40 (0.54)	0.20	0.15	77
GAD7	8.10 (5.16)	6.93	9.28	0.45 (0.27)	−0.57 (0.54)	0.18	0.02	77
PIH	32.83 (17.95)	28.76	36.90	0.27 (0.27)	−0.49 (0.54)	0.20	0.13	77
WHOQOL‐BREF	62.94 (13.54)	59.86	66.01	−0.08 (0.27)	−0.56 (0.54)	0.20	0.54	77

Abbreviations: CES‐D, Center for Epidemiologic Studies Depression; GAD7, General Anxiety Disorder 7; PIH, Partners in Health; WHOQOL‐BREF, World Health Organization Quality of Life Bref.

Population averages were compared: for the PIH, this sample mean (*M* = 32.83, SD = 17.95) reported a higher score (worse self‐management) than the population average (*M* = 23.6, SD = 12.9); for the GAD‐7, this sample mean (*M* = 8.10, SD = 5.16) reported a higher score (higher anxiety) than the population average (*M* = 4.9, SD = 4.8); and for the CES‐D scale, this sample mean (*M* = 22.49, SD = 11.27) reported a higher score (higher depression) than the population average (*M* = 9.25, SD = 8.58). Scatterplots and correlations between variables (see Table 3) were analysed. No correlation was found between adjacent residuals (Durbin–Watson statistic = 2.07), and the Variance Inflation Factor revealed that multicollinearity is not likely to be an issue (see Table 4).

**TABLE 3:** Pearson correlation coefficients (and significance levels) for PIH, GAD7, CES‐D and WHOQOL‐BREF scores

	WHOQOL‐BREF	PIH	GAD7	CES‐D
WHOQOL‐BREF	1.00	−0.20 (0.04)	−0.43 (0.00)	−0.65 (0.00)
PIH		1.00	0.26 (0.01)	0.39 (0.00)
GAD7			1.00	0.78 (0.00)
CES‐D				1.00

Abbreviations: CES‐D, Center for Epidemiologic Studies Depression; GAD7, General Anxiety Disorder 7; PIH, Partners in Health; WHOQOL‐BREF, World Health Organization Quality of Life Bref.

**TABLE 4:** Unstandardized beta values (*B*), standard errors of unstandardized beta values (SE *B*), standardized beta coefficients (*β*), and VIF for PIH, GAD7, CES‐D and WHOQOL‐BREF scores

	*B*	SE *B*	*β*	VIF
Constant	67.90	3.19		
PIH	−0.15	0.09	−0.20	1.00
Constant	73.94	3.34		
PIH	−0.07	0.08	−0.09	1.07
GAD7	−1.07	0.28	−0.41	1.07
Constant	79.36	2.99		
PIH	0.05	0.07	0.07	1.19
GAD7	0.51	0.37	0.20	2.60
CES‐D	−0.99	0.18	−0.83	2.86

Abbreviations: CES‐D, Center for Epidemiologic Studies Depression; GAD7, General Anxiety Disorder 7; PIH, Partners in Health; VIF, variance inflation factor.

Hierarchical multiple linear regression across three models analysed for correlation between the PIH (self‐management), GAD7 (anxiety) and CES‐D (depression) scores, and the WHOQOL‐BREF (quality of life) score. Model 1 showed no significant variance contributed by PIH scores to quality of life scores (*R* ^2^ = 0.04, *R* ^2^_Adj_ = 0.03, *p* = 0.08). However, Model 2 showed that PIH scores and GAD7 scores combined account for 17.4% of the variance in quality of life scores (*R* ^2^ = 0.20, *R* ^2^_Adj_ = 0.17, *p* < 0.001), and in Model 3 the combined PIH, GAD7 and CES‐D scores account for 41.1% of the variance in quality of life scores (*R* ^2^ = 0.43, *R* ^2^_Adj_ = 0.41, *p* < 0.001). These results show that self‐management of chronic eczema, anxiety, and depression combined significantly predict quality of life (*F*_(3, 73)_ = 18.68, *p* < 0.001).

In Model 2, there was a significant negative relationship between anxiety and quality of life scores (*t*_(76)_ = −3.79, *p* < 0.001), with an increase of one unit in anxiety score predicting a decrease of 1.07 units in quality of life score. In Model 3, there was also a significant negative relationship between depression and quality of life scores (*t*_(76)_ = −5.55, *p* < 0.001), with an increase of one unit in depression score predicting a decrease of 0.99 units in quality of life score. However, Model 1 and all other variables in Models 2 and 3 were not significant predictors.

In addition, considering only the PIH questions associated with factors 1 (core self‐management) and 2 (condition knowledge), Model 1 shows significance, accounting for 4% of variance in quality of life (*R* ^2^ = 0.06, *R* ^2^_Adj_ = 0.04, *p* = 0.04), and the combined scales in Model 3, using only factors 1 and 2 of the PIH, are a significant predictor of quality of life (*F*_(3, 73)_ = 18.51, *p* < 0.001). Furthermore, in Model 1 (PIH factors 1 and 2), there was a significant negative relationship between self‐management and quality of life scores (*t*_(76)_ = −2.08, *p* = 0.04), with an increase of one unit in self‐management score predicting a decrease of 0.22 units in quality of life score (see Tables 5 and 6).

**TABLE 5:** Pearson correlation coefficients (and significance levels) for PIH (factors 1 and 2), GAD7, CES‐D and WHOQOL‐BREF scores

	WHOQOL‐BREF	PIH (F 1 & 2)	GAD7	CES‐D
WHOQOL‐BREF	1.00	−0.23 (0.02)	−0.43 (0.00)	−0.65 (0.00)
PIH (F 1 and 2)		1.00	0.28 (0.01)	0.41 (0.00)
GAD7			1.00	0.78 (0.00)
CES‐D				1.00

Abbreviations: CES‐D, Center for Epidemiologic Studies Depression; GAD7, General Anxiety Disorder 7; PIH, Partners in Health; VIF, variance inflation factor; WHOQOL‐BREF, World Health Organization Quality of Life Bref.

**TABLE 6:** Unstandardized beta values (*B*), standard errors of unstandardized beta values (SE *B*), standardized beta coefficients (*β*), and VIF for PIH (factors 1 and 2), GAD7, CES‐D, and WHOQOL‐BREF scores

	*B*	SE *B*	*β*	VIF
Constant	68.80	3.20		
PIH (F 1 and 2)	−0.22	0.10	−0.23	1.00
Constant	74.51	3.34		
PIH (F 1 and 2)	−0.11	0.10	−0.12	1.08
GAD7	−1.07	0.28	−0.40	1.08
Constant	79.70	2.99		
PIH (F 1 and 2)	0.04	0.09	0.05	1.21
GAD7	0.51	0.37	0.19	2.61
CES‐D	−0.98	0.18	−0.82	2.90

Abbreviations: CES‐D, Center for Epidemiologic Studies Depression; GAD7, General Anxiety Disorder 7; PIH, Partners in Health; VIF, variance inflation factor.

Post‐hoc exploratory analysis of means plots revealed that increasing education levels show a trend with improved self‐management. There is a similar trend in increasing quality of life scores between secondary education and postgraduate education in this sample.

A post‐hoc power analysis revealed this study to have high power (0.80), which supports that no Type‐II error has occurred.^36^

## 6 DISCUSSION

This study aimed at assessing for the influence of eczema self‐management, anxiety, and depression on eczema‐related quality of life. The hypotheses anticipated that high self‐management with low anxiety and depression would positively influence quality of life; that low self‐management with high anxiety and depression would negatively influence quality of life; and that high anxiety or depression would reduce the influence of self‐management of chronic eczema on quality of life.

A hierarchical multiple linear regression across three models revealed that self‐management and anxiety together significantly predict quality of life, with anxiety showing a negative correlational relationship, whereby anxiety is associated with lower quality of life; and that self‐management, anxiety, and depression together significantly predict quality of life, with depression showing a negative correlational relationship, whereby depression is associated with lower quality of life. A further hierarchical multiple linear regression assessed only self‐management data associated with the factors of core self‐management and condition knowledge, alongside anxiety and depression, which revealed that these factors alone significantly predict quality of life, showing a negative correlational relationship.

These findings suggest self‐management influences quality of life.^16^, ^18^ However, the negative correlation between anxiety and quality of life becomes more statistically relevant than self‐management. One study^37^ shows that self‐management of eczema (*p* < 0.014) influences quality of life less than GAD7 anxiety scores (*p* < 0.001) alongside eczema reported by another study.^9^ Furthermore, depression reduces the significance of both self‐management and anxiety. A recent systematic literature review and meta‐analysis^27^ support that depression is more significantly associated with eczema than anxiety. Importantly, anxiety more frequently presents as a comorbidity with eczema than depression.^2^, ^4^, ^7^, ^22^

Self‐management positively correlates with quality of life,^14^, ^17^, ^19^ and the current findings focus on chronic eczema. Furthermore, the current sample shows a trend that as education level increases, self‐management understanding increases. Individuals with the ability to self‐manage eczema are more likely to support their own treatment,^15^, ^38^ and one study shows potential lessening of symptoms in those with hand eczema.^5^ Previous research shows that individuals with anxiety or depression are less likely to engage in self‐management, which can worsen symptoms^25^, ^39^ associated with decreasing quality of life.^3^, ^9^ In this sample, self‐management alone significantly predicts quality of life; but anxiety and depression decrease the strength of the predicting relationship between self‐management and quality of life.

It is important to note that each variable contributes variance to the outcome variable of quality of life, showing a meaningful trend, even though not all achieve statistical significance, which might have clinical relevance.^40^ Furthermore, no causal relationship is apparent.^40^, ^41^ Of interest is that the current sample means for self‐management, anxiety and depression reported in this study showed worse self‐management and higher anxiety and depression than in respective population averages.^12^, ^32^, ^33^ This might reflect potential clinical reluctance to offer public education around eczema self‐management, and possibly a lack of diagnosis of anxiety and depression.^42^ This may result in worsening eczema symptoms, negatively influencing quality of life in this sample.

### 6.1 Implications

The findings of this study suggest eczema self‐management education has the potential to lessen symptoms and influence quality of life, in particular, shared decision‐making, attending appointments, medication adherence, responding to symptom progression, healthy lifestyle choices and condition knowledge.^12^ In addition, these findings contribute to the growing body of evidence^7^, ^9^, ^27^ that anxiety and depression influence health‐related quality of life, and also suggest a reduction in the influence of eczema self‐management.

### 6.2 Strengths and limitations

Strengths of this study include the use of an attention check question and the high reliability and validity of the four scales presented in randomized order.^40^

A limitation of the current study is the lack of a control group. A recent study^9^ on individuals with atopic eczema found a significant difference in a control group without eczema; however, the demographics did not match the eczema‐condition demographics. Furthermore, limited available psychological scales assess for self‐management of eczema. In particular, the mean age (66 years) of the tested demographics for the PIH scale^12^ were markedly different to the predominant age range (18–29 = 77%) of the current study.

It is also important to acknowledge that this study was opportunistically recruited, self‐reported online, not allowing for medical verification of a chronic eczema diagnosis, and the participants' environment was uncontrolled; although, as the authors of the PIH scale^12^ note, objective interviewer responses strongly match participant responses. Therefore, the high reliability and validity ratings of the four scales ensure robust data.

### 6.3 Future research

Future research might consider severity of eczema symptoms, anxiety or depression. Recent studies have found that greater severity of eczema symptoms increases the risk of anxiety and depression^7^, ^22^, ^43^ and decreases quality of life.^10^, ^19^ Furthermore, increasing levels of anxiety and depression increase eczema symptom severity.^20^ However, these have not been considered in conjunction with self‐management. Furthermore, the PIH interviewer cue and response form^12^ might allow for a qualitative understanding of these findings. Similarly, longitudinal research might offer further insight.^44^

## 7 CONCLUSION

In conclusion, the aim of this study was to establish whether anxiety and depression influence self‐management of chronic eczema in relation to quality of life. The findings suggest that self‐management, anxiety and depression all influence quality of life, and that the presence of comorbid anxiety and depression with chronic eczema decreases the contributed variance of self‐management in this sample. This has important implications for primary care treatment of chronic eczema, as self‐management education might be less effective when patients experience symptoms of anxiety or depression.

## CONFLICT OF INTEREST

The authors have no competing interests to report.

## ETHICS STATEMENT

Ethical approval was granted by the University of Derby Research Ethics Committee. All ethical considerations were taken into account with regards the British Psychological Society (BPS) Standards and Requirements,^32^ the BPS Ethics Guidelines for Internet‐Mediated Research,^33^ the University of Derby Code of Ethics,^34^ and General Data Protection Regulation.^35^

## AUTHOR CONTRIBUTIONS

**Laura Standen:** Conceptualization (lead); data curation (lead); formal analysis (lead); investigation (lead); methodology (lead); project administration (lead); writing – original draft (lead); writing – review & editing (equal). **Gulcan Garip:** Conceptualization (supporting); data curation (supporting); formal analysis (supporting); investigation (supporting); methodology (supporting); supervision (lead); writing – original draft (supporting); writing – review & editing (equal).

## Data Availability

Data available on request from the authors.
